# Uncertainty principle of genetic information in a living cell

**DOI:** 10.1186/1742-4682-2-40

**Published:** 2005-09-30

**Authors:** Pierluigi Strippoli, Silvia Canaider, Francesco Noferini, Pietro D'Addabbo, Lorenza Vitale, Federica Facchin, Luca Lenzi, Raffaella Casadei, Paolo Carinci, Maria Zannotti, Flavia Frabetti

**Affiliations:** 1 Center for Research in Molecular Genetics "Fondazione CARISBO", Department of Histology, Embriology and Applied Biology, University of Bologna, Via Belmeloro 8, 40126 Bologna (BO), Italy; 2 Department of Physics, University of Bologna, Via Irnerio 46, 40126 Bologna (BO), Italy; Sezione INFN, Bologna, Italy; 3 Dipartimento di Genetica e Microbiologia, University of Bari, 70126 Bari, Italy

## Abstract

**Background:**

Formal description of a cell's genetic information should provide the number of DNA molecules in that cell and their complete nucleotide sequences. We pose the formal problem: can the genome sequence forming the genotype of a given living cell be known with absolute certainty so that the cell's behaviour (phenotype) can be correlated to that genetic information? To answer this question, we propose a series of thought experiments.

**Results:**

We show that the genome sequence of any actual living cell cannot physically be known with absolute certainty, independently of the method used. There is an associated uncertainty, in terms of base pairs, equal to or greater than μs (where μ is the mutation rate of the cell type and s is the cell's genome size).

**Conclusion:**

This finding establishes an "uncertainty principle" in genetics for the first time, and its analogy with the Heisenberg uncertainty principle in physics is discussed. The genetic information that makes living cells work is thus better represented by a probabilistic model rather than as a completely defined object.

## Background

### The formal problem of knowing the genome sequence in a living cell

**Figure 1:**
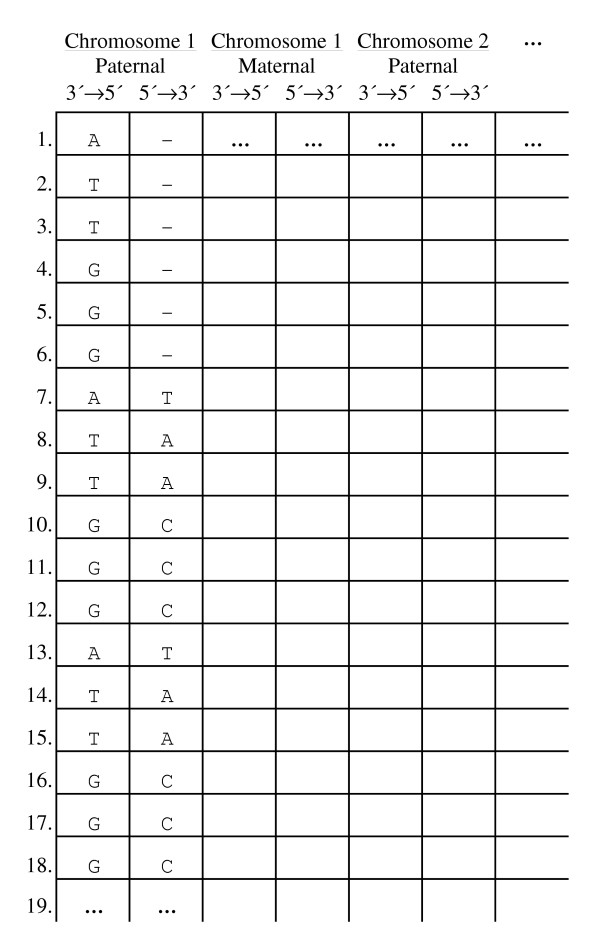
Formal representation of total cellular genetic information. Each matrix column should contain the sequence of each distinct DNA molecule strand in the cell (e.g. human sequence data), because mutations first arise only in one strand, and telomeres normally have a protruding single-strand of variable length.

We pose the formal problem: can the genome sequence forming the genotype of a given living cell be known with absolute certainty so that the cell's behaviour (phenotype) can be correlated to that genetic information? Firstly, the genome being the cell's DNA content [1], we define the description of the total genetic information "I" (the cell's genome sequence, forming its genotype) as a matrix comprising the linear base sequences for the distinct genomic DNA molecules in that cell (Fig. 1). For the purpose of this discussion, a living cell (prokaryotic or eukaryotic, from a monocellular or multicellular organism, germinal or somatic) is able to perform all its normal natural functions (operatively, capacity for division and/or development into an organism, and/or performance of the functions typical of its terminally differentiated state). A consensus sequence is a sequence created by choosing, for each position, the most representative base in a set of aligned DNA sequences. It should be noted that all genomic sequences provided by modern genome projects (e.g. human) [2,3] are actually consensus sequences for different homologous chromosomes (in the case of diploid cells), different cells [4], and, often, different individuals. It is worth emphasizing that there is no formal proof that such a "mean" sequence would work in a real cell. Furthermore, each living cell experiences continuous progression from one state, i.e. a pattern configuration of the system at a particular instant, to another [5], and even in a non-dividing cell the genome structure is subjected to dynamic changes over time due to DNA modifications, lesions and repair [6]. However, for the purpose of discussing the problem posed above, we assume the existence of a completely defined cell genomic DNA sequence that is determined at a certain "time zero" instant.

We propose three thought experiments to show how "I" could be determined with absolute certainty in a living cell, assuming that, after determination of the genome sequence, the original cell is further available for tracing its behaviour, simulating or verifying predictions about its genotype/phenotype relationships, or obtaining derivative cells or organisms.

The most common method used is to isolate the cell's DNA molecules and sequence them by enzymatic or chemical manipulations. In the case of a single cell, several technical problems must be faced: it is difficult to extract the very small amount of DNA without damaging it, and the requisite in vivo or in vitro amplification of the molecules may add artifactual mutations. However, for the purpose of this discussion, we hypothesize that a suitable method could be devised. Even in this case, however, knowledge of "I" would coincide with the irreversible unavailability of the original cell to exploit that biological information.

An alternative to traditional DNA sequencing could be direct imaging of the DNA molecules, at a level of resolution sufficient to read its sequence. In principle, this method could be extended to reading the DNA sequence inside a living cell ("Star Trek" method) [7]. By definition, the wavelength used to image the DNA sequence would have to be adequate for resolution in the order of the atomic radius (~0.1 nm), so high frequency and energy (>10 keV) are physically inevitable. If a single cell were irradiated with >10 keV waves in order to image each segment of the millions or billions of base pairs constituting its DNA (10^-9^–10^-6 ^J absorbed, respectively, even hypothesizing one particle for each base pair) it could not survive this irradiation, which is several orders of magnitude greater than the lethal dose (~1000 rad [8] = 10 Gy, i.e. ~10^-11 ^J/ng). In addition, it has recently been demonstrated that secondary free electrons, even at energies well below ionization thresholds, induce single- and double-strand breaks in DNA [9], thus in any case modifying the original genetic information "I" in the cell.

Scanning probe microscopes are based on a new concept of very high-resolution imaging, and they are being studied as a method for DNA sequencing [10]. Although they do not use high-energy radiation, these instruments deploy a microscopic tip that scans the molecule surface from very close range. Their suitability for DNA sequencing depends critically on the successful preparation of DNA on a surface [10], which is again not consistent with the maintenance of cell integrity.

A different method for deriving the sequence of a DNA molecule based on assessment of its energetic state, without needing to "visualize" its molecular shape, has been discussed on purely theoretical grounds [11]. It has been shown that an uncertainty relationship emerges between temperature and the order (negative entropy) of the DNA molecule [11]. This makes it impossible to reach absolute certainty about the structure of the DNA, even if this method should become technically feasible and shown to be applicable to DNA in living cells.

**Figure 2:**
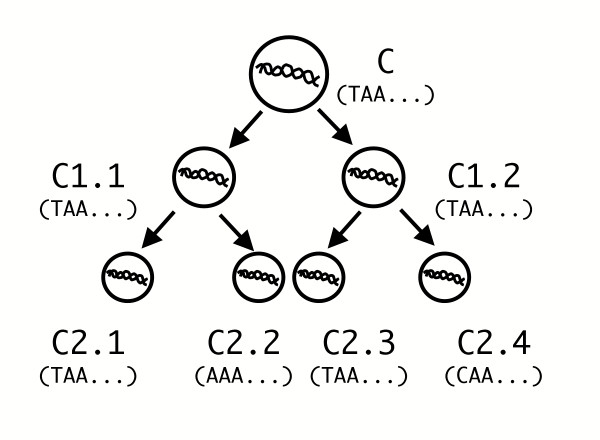
Determination of total genetic information of a cell genome: nearest relative analysis; in this case, even sequence identity among multiple cells from a common ancestor "C" (e.g. C2.1 and C2.3) is not formal proof of sequence identity with the other extant cells (e.g. C2.2 and C2.4). For simplicity, only the sequence of one strand is shown.

The only remaining method appears to be genome sequencing of a cell with supposedly identical genetic information. This procedure will destroy the test cell, leaving an equivalent living cell available for observation. The most adequate test cell would be a direct relative of the cell to be studied (Fig. 2). However, any cell is separated from its nearest relative by at least one cell division. In this process, a copy of the genome is made and each copy is distributed to the two daughter cells. The DNA replication process is central to cell life, and it is accomplished by complexes of copying and proofreading enzymes. These proteins are molecular machines subject to the laws of thermodynamics [12], and their effectiveness cannot be 100 percent; thus, replication errors inevitably accumulate during successive cell divisions [13,14]. These errors lead to changes in the original sequence (mutations, including polymorphisms and pathogenic mutations), and the "mutation rate" for a given organism can be defined as the number of changed positions (in base pair, bp) for each cell per generation [14]. The mutation rate is, in nature, greater than 0, so in each cell a certain number of base pairs is likely to differ from those in the initial genome.

## Results and Discussion

### Uncertainty principle of genetic information in a living cell

In view of the above-described thought experiments, we conclude that in a genome of total size "s" (measured in bp), the average number of mutated base pairs, used as a measure of uncertainty (U) about its actual sequence in a living cell, can be quantified by:

U ≥ μs     (1)

where μ is the mutation rate of the cell type under consideration. For example, in the human genome, uncorrected replication errors occur with a frequency varying between 10^-9 ^and 10^-11 ^per incorporated nucleotide [14], depending in particular on the type of genome region [15,16]. Considering the total length of the human genome sequence (~6 × 10^9 ^bp), the overall uncertainty in the identity of the whole sequence is between 6 and 0.06 nucleotides per replication, meaning in the latter case that one cell will have a probability of 6 percent of having one mutation per replication. For simplicity, we do not consider other possible but less frequent contributions to overall mutation deriving from the distribution, rather than replication, of nuclear or mitochondrial DNA molecules [14].

It should also be noted that any conceivable method for measuring the incorporation of nucleotides to determine the actual sequence in a living cell will similarly entail an error proportional to the mutation rate, because the accuracy of any such method is ultimately dependent on the accuracy of the DNA replication machinery.

In the case of stem cell replication, it is possible that the same original "immortal strand" is continuously retained by an undifferentiating stem cell, while the newly synthesized strand is asymmetrically distributed, at the next cell replication, to the differentiating daughter cell [17]. In this selected case the sequence of a stem lineage cell (e.g. cells C, C1.1 and C2.1 in Fig. 2) could be derived from the consensus sequence from randomly mutated differentiating daughter cells (e.g. C1.2, C2.2 and so on in Fig. 2). However, at each moment, the stem cell also retains a newly synthesized and potentially mutated strand, the sequence of which can only be known with an associated uncertainty that is, again, proportional to the mutation rate. This does not allow the matrix in Fig. 1 to be completed with absolute certainty for that cell.

The actual genome sequence in any living cell can thus be known only with a certain amount of indeterminacy, which may be very small but is always greater than 0 because of fixed physical constraints dictated by the cell structure itself and by formal limits on any process for determining DNA sequences without disrupting the cell. These limits are in turn intrinsically related to the submicroscopic scale of genetic information in nature, independently of any methodological approach or any current or future technological device. The importance of any single base pair for the phenotype cannot be over-emphasized, as exemplified, for example, by the case of human achondroplasia (short-limb dwarfism), in which a single base substitution in a single chromosome invariably has dramatic effects on skeleton growth [18] via a single amino acid change.

In addition, there is growing evidence that genomic regions other than classical gene protein-coding regions have biological function. Changes in the 5' or 3' untranslated regions of mRNAs have been recently related to disease phenotypes [e.g. [19,20]]. Many types of functional "noncoding" RNAs [21] may be transcribed from non-genic regions or from the opposite DNA strand in protein-coding genes, even in classical constitutive heterochromatin zones. For instance, yeast centromeric repeat sequences have recently been shown to be transcribed and then processed by components of the RNA interference (a sequence-specific gene silencing) pathway [22]. Finally, even mutations in coding regions previously deemed "silent" (mutations that do not affect the amino acid sequence) may have phenotypic effects via their influence on splicing accuracy or efficiency [23]. In general, organisms with larger genome sizes tend to have a greater number of deleterious mutations, and it has been estimated that, in humans, the deleterious genomic mutation rate is high [24]; it should also be noted that many phenotypic changes induced by variations in a particular genomic region could be present but could go undetected if they do not grossly affect morphology and physiology and if they are not directly, actively searched. Overally, this information clearly indicates that the relevance of small numbers of subtle mutations in a single cell may be high, particularly if this cell is the founder of a new organism or a new colony of individuals. Thus, although the connectivity of networks between genes and transcription factors and the complexity achieved by genetically encoded information-processing systems such as nervous and immune systems add further dimensions to biological complexity [25], it is important to establish whether the genetic information of a living cell may be known definitely in its entirety.

The uncertainty principle discussed here should not be confused with the critique of biological determinism, which states that, given a certain piece of biological information, we cannot confidently predict the behaviour of the whole cell or organism because of the complex relationships between genotype and phenotype [26]. Uncertainty has been also proposed in biology in respect of the full understanding of gene function. Owing to effects of gene function that are possibly important for long-term fitness within a population but very small in individuals, the formal elucidation of gene function could require experiments on an evolutionary scale, involving the whole population of the relevant species [27]. Finally, a purely qualitative uncertainty relationship has been put forward between the degree of molecular perturbation in the cells investigated and the number of biological pathways simultaneously examined by the "array" approach (able to monitor genome-wide DNA expression profiles) [28]. In these and similar discussions it is assumed that the cell genome is a known starting point and the problem lies in predicting how epigenetic changes (DNA modifications that can alter gene expression without changing DNA sequences), RNA editing (post-transcriptional RNA modification), post-translational protein modification or any other intracellular or extracellular interacting factor might affect the expression of genetic information.

Our concept applies upstream of these problems: defining intrinsic uncertainty in the knowledge of a complete, actual genotype, to be further related to a phenotypic/functional outcome. This type of uncertainty also reinforces arguments against the reductionist approach to biology, i.e. the attempt to explain complex phenomena by listing all the individual components of multicomponent systems and defining their functional properties [29]. Systems biology has recently emerged as the successor to reductionism, seeking to predict the behaviour or "emergent properties" of complex, multicomponent biological processes by trying to understand the general picture rather than the sum of the workings of the parts in isolation [29]. Although systems biology could cope with indeterminacy in the formal knowledge of the complete cell "parts list", including its complete genome sequence, its models always remain subject to an irreducible degree of unpredictability due to the sum of intractable uncertainties at each successive level of investigation from genes to the whole organism.

Possible practical implications of the uncertainty principle of genetic information in a living cell concern problems such as in silico cell modeling and the diagnostic value of specific methods. These implications will need further specific investigation and discussion.

### Genomics and the physical limits of the knowledge

We have presented here the first uncertainty principle to be announced in structural genomics. This is an addition to the uncertainty principles in physics, where Heisenberg established that it is impossible to know the position and the momentum of an electron simultaneously with absolute certainty (Heisenberg's uncertainty principle) [30], and in mathematics, where Gödel showed that a great variety of logical systems contain formally undecidable propositions [31].

In the broadest sense, statements of this type all demonstrate the formal impossibility of knowing a given system at a desired arbitrary level [32], although in his 1927 article Werner Heisenberg insisted that the uncertainty he described is not due to technical or intrinsic features of the measuring process, but it is a fundamental feature of reality itself, i.e. an electron cannot in principle have a precise position and momentum simultaneously. It is interesting to note that in his 1933 lecture "Light and life" [33], Niels Bohr applied an analogous uncertainty concept in biology to argue that a living being would be killed by detailed physical investigation, so there is "complementarity" between the simultaneous existence of life and the possibility of describing it scientifically. Bohr concluded that life "must be considered an elementary fact that cannot be explained" (although in his later 1962 revisitation of the problem [34] he avoided any reference to incompatibility between scientific description and existence of life, possibly influenced by results in molecular biology obtained by his student Max Delbrück [35]). In our case, instead, uncertainty arises from the intrinsic impossibility of determining a physical quantity that nevertheless exists (the real genome sequence present at a given instant within a living cell).

However, if we consider the evolution of the state of a system, the analogy may still hold: in physics, the Heisenberg principle affects any attempt to determine the future behaviour of an atomic particle in a certain position; in genetics, the future biological behaviour of a living cell cannot be linked with absolute certainty to the positions of nucleotides in the current genome sequence. For a living cell, we can only determine a "consensus" sequence from its relatives, and this fluctuates with a certain probability around the actual sequence. Recently, the concept that an ideal "average cell" exists has been challenged in respect of gene expression, and it has been shown that, although expression at the cellular level does not require tight specifications and there is high tolerance of variation, each single nucleus is probabilistic in its expression repertoire [36].

Finally, we note that replication errors leading to spontaneous point mutations arise from transient alternative states of the DNA base functional groups (tautomeric shifts [37], base ionization [38]). Precise knowledge of the quantum jump events in the base molecule could allow subsequent copy errors to be predicted [39,40], but the Heisenberg principle does not allow this with complete certainty. In this sense, the Heisenberg principle is not only analogous to the genetic information uncertainty principle, but is profoundly relevant to the roots of the latter.

## Competing interests

The author(s) declare that they have no competing interests.

## Authors' contributions

All authors contributed to define the concept that we present; they all drafted the manuscript and approved the final version.
